# Supplementary material for: Direct detection of coupled proton and electron transfers in human manganese superoxide dismutase
Source: Nat Commun. 2021 Apr 6;12:2079. doi: 10.1038/s41467-021-22290-1 (PMC8024262; doi:10.1038/s41467-021-22290-1)
Supplement: Supplementary file 1 — Supplementary Information [file 41467_2021_22290_MOESM1_ESM.pdf]

Supplementary Information

Direct detection of coupled proton and electron transfers in human manganese superoxide dismutase

Jahaun Azadmanesh[1], William E. Lutz[2], Leighton Coates[3], Kevin L. Weiss[4], and Gloria E. O. Borgstahl[1,2]*

[1]Department of Biochemistry and Molecular Biology, 985870 Nebraska Medical Center, Omaha, NE 68198-5870, USA

[2]Eppley Institute for Cancer and Allied Diseases, 986805 Nebraska Medical Center, Omaha, NE 68198-6805, USA

[3]Second Target Station, Oak Ridge National Laboratory, 1 Bethel Valley Road, Oak Ridge, TN 37831, USA

[4]Neutron Scattering Division, Oak Ridge National Laboratory, 1 Bethel Valley Road, Oak Ridge, TN 37831, USA

*gborgstahl@unmc.edu

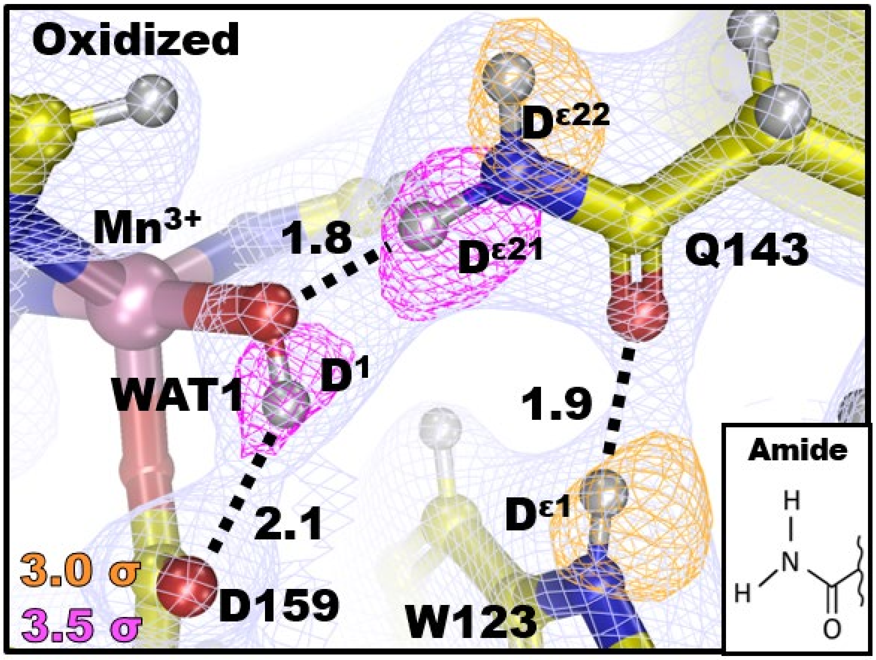

Supplementary Figure 1. **Neutron structure at the active site of Mn^{3+}SOD chain A.** Magenta and orange omit $|F_o|$-$|F_c|$ difference neutron scattering length density is displayed at 3.5σ and 3.0σ, respectively, and light blue 2$|F_o|$-$|F_c|$ neutron scattering length density is displayed at 1.0σ.

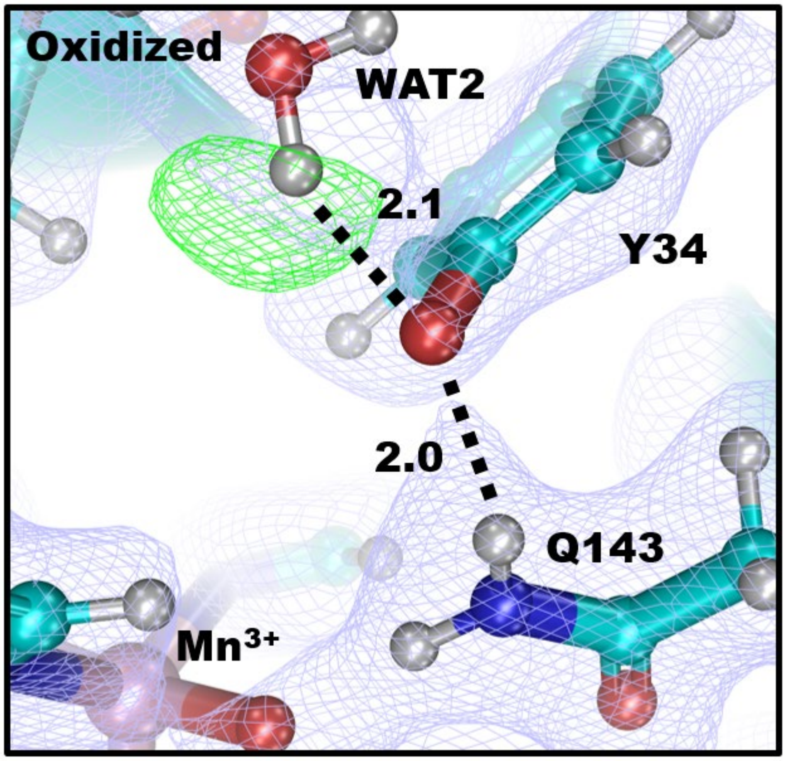

Supplementary Figure 2. **Neutron structure at the active site of Mn^{3+}SOD chain B.** Green omit $|F_o|$-$|F_c|$ difference neutron scattering length density is displayed at 2.5σ and light blue 2$|F_o|$-$|F_c|$ neutron scattering length density is displayed at 1.0σ.

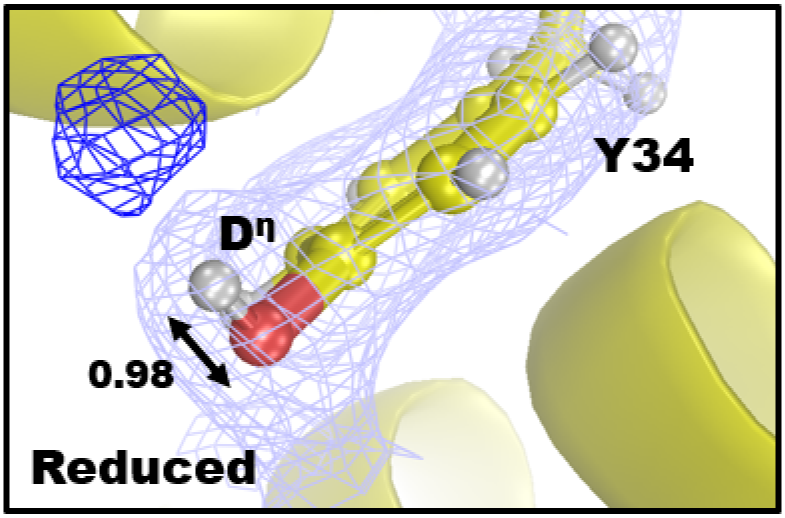

Supplementary Figure 3. **Residual density for the hydroxyl group of Tyr34 in Mn^{2+}SOD of chain B.** Light blue 2|F_o|-|F_c| neutron scattering length density displayed at 1.0 σ. Dark blue omit |F_o|-|F_c| difference density is displayed at 2.0 σ. Bond length in Å is given.

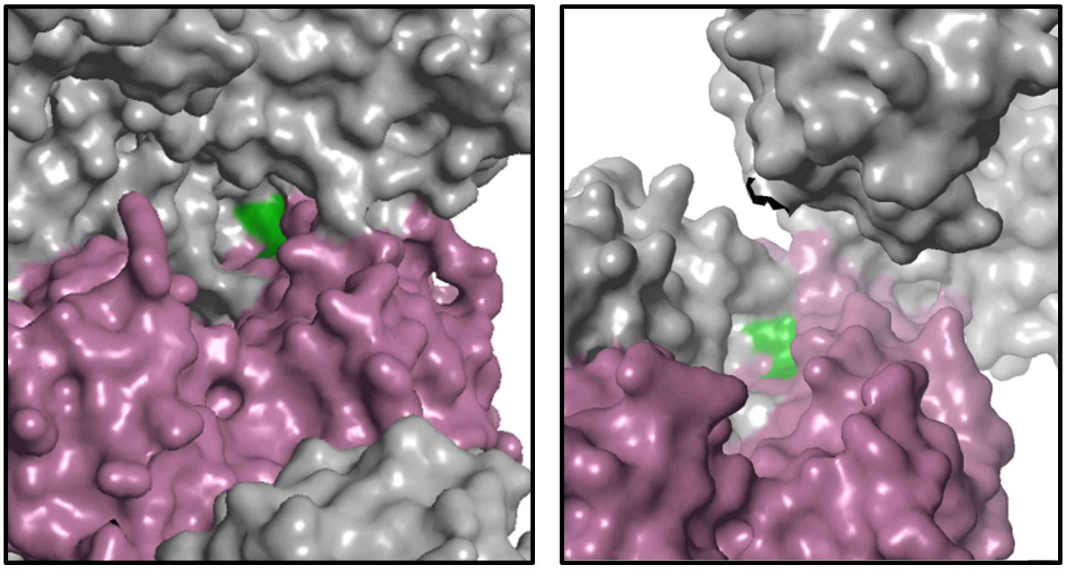

Chain A **Chain B**

Supplementary Figure 4. **Solvent accessibility differences between chains of the asymmetric AB dimer for $P6_{1}22$ MnSOD.** Magenta depicts the surfaces of the chain for the asymmetric unit and green depicts the surfaces leading to the active site of the chain. Grey depicts the surfaces of symmetry generated asymmetric AB dimers.

Supplementary Table 1. **Gln143 bonding character from CLPO analysis.** Numbers are calculated bond order.

	Five-Coordinate Mn^{3+} Y166(-)H30(ε)Y34(-)	Five-Coordinate Mn^{3+} Y166(H)H30(δ)Y34(-)	Six-Coordinate Mn^{2+} Y166(H)H30(δ)Y34(-)	Five-Coordinate Mn^{2+} Y166(H)H30(δ)Y34(H)
$N^{\varepsilon 2}$-$C^{\varepsilon 1}$	1.36	1.37	1.56	1.52
$O^{\varepsilon 1}$-$C^{\varepsilon 1}$	1.45	1.45	1.30	1.33

Supplementary Table 2. **Charge and energy interactions of donor-acceptor CLPO analysis.**

Trp123 $N^{\varepsilon 1}$ Lone Pair → Trp123 $C^{\varepsilon 2}$-$C^{\delta 2}$ π^*-bond				
State	Donor Occupancy (e^-)	Charge Transfer (e^-)	Acceptor Occupancy (e^-)	Energy Stabilization (kcal mol^{-1})
Five-Coordinate Mn^{2+} Y166(H)H30(δ)Y34(H)	1.58	0.15	0.52	↓ 13.52
Gln143 $N^{\varepsilon 2}$ Lone pair → WAT1 O-H σ^*-bond				
State	Donor Occupancy (e^-)	Charge Transfer (e^-)	Acceptor Occupancy (e^-)	Energy Stabilization (kcal mol^{-1})
Six-Coordinate Mn^{2+} Y166(H)H30(δ)Y34(-)	1.81	0.14	0.15	↓ 0.46
Five-Coordinate Mn^{2+} Y166(H)H30(δ)Y34(H)	1.78	0.17	0.18	↓ 1.39

Supplementary Table 3. **Percent covalence of shared hydrogen atoms in SSHBs bonds from CLPO analysis.**

	Five-Coordinate Mn^{3+} Y166(-)H30(ε)Y34(-)		Five-Coordinate Mn^{3+} Y166(H)H30(δ)Y34(-)		Six-Coordinate Mn^{2+} Y166(H)H30(δ)Y34(-)		Five-Coordinate Mn^{2+} Y166(H)H30(δ)Y34(H)	
(Gln143)$N^{\varepsilon 2}$-H-O(WAT1)	$N^{\varepsilon 2}$	O	$N^{\varepsilon 2}$	O	$N^{\varepsilon 2}$	O	$N^{\varepsilon 2}$	O
	0.92	0.08	0.92	0.08	0.29	0.71	0.36	0.64
(Tyr166)O^{η}-H-$N^{\varepsilon 2}$(His30)	O^{η}	$N^{\varepsilon 2}$	O^{η}	$N^{\varepsilon 2}$	O^{η}	$N^{\varepsilon 2}$	O^{η}	$N^{\varepsilon 2}$
	0.20	0.80	0.77	0.23	0.76	0.24	0.79	0.21
(Tyr34)O^{η}-H-O(WAT2)	O^{η}	O	O^{η}	O	O^{η}	O	O^{η}	O
	0.15	0.85	0.23	0.77	0.20	0.80	0.90	0.10
(His30)$N^{\delta 1}$-H-O(WAT2)	$N^{\delta 1}$	O	$N^{\delta 1}$	O	$N^{\delta 1}$	O	$N^{\delta 1}$	O
	0.14	0.86	0.89	0.11	0.88	0.12	0.92	0.08

Supplementary Table 4. **Active Site B-factors (Å^2) of MnSOD Neutron Structures.**

		Mn^{3+}SOD		Mn^{2+}SOD	
Molecule	Atom	A	B	A	B
WAT1	O	22.8	30.92	18.4	20.2
	D^1	27.3	36.99	21.8	23.9
	D^2	-	-	-	23.9
Q143	$N^{\varepsilon 2}$	23.1	21.4	17.8	27.2
	$D^{\varepsilon 21}$	27.3	37.0	[b]21.8	-
	$D^{\varepsilon 22}$	27.6	25.6	21.1	32.3
Y34	O^{η}	20.2	27.2	25.2	21.9
H30	$N^{\delta 1}$	24.9	17.8	29.9	17.1
	$D^{\delta 1}$	29.8	-	35.6	20.3
	$N^{\varepsilon 2}$	24.1	18.7	23.5	17.7
Y166	O^{η}	26.1	23.1	21.7	19.7
[a]H30/Y166	$D^{\varepsilon 2}/D^{\eta}$	28.8	22.3	28.0	20.9

[a]D atom between $N^{\varepsilon 2}$(H30) and O^{η}(Y166). For Mn^{3+}SOD, the atom is closest to O^{η}(Y166). For Mn^{2+}SOD, the atom is equidistant between $N^{\varepsilon 2}$(H30) and O^{η}(Y166).

[b]For chain A of Mn^{2+}SOD, $D^{\varepsilon 21}$(Q143) is 1.4 Å from O(WAT1) and may be partially bonded with it.

Supplementary Table 5. **Active Site Bond Lengths of MnSOD Neutron Structures.**

	Mn^{3+}SOD		Mn^{2+}SOD	
Mn Covalent Bonds (Å)	A	B	A	B
Mn-$N^{\varepsilon 2}$(H26)	2.07	2.07	2.26	2.10
Mn-$N^{\varepsilon 2}$(H74)	2.13	2.12	2.19	2.25
Mn-$O^{\varepsilon 2}$(D159)	1.95	1.94	2.44	2.15
Mn-$N^{\varepsilon 2}$(H163)	2.06	2.14	2.23	2.21
Mn-O(WAT1)	1.78	1.76	2.12	2.22
Mn-O(OL)	-	-	1.82	-

Supplementary Table 6. **Data collection and refinement statistics**

Data Collection Statistics				
	Neutron		X-ray	
	Oxidized	Reduced	Oxidized	Reduced
PDB Code	7KKS	7KKW	7KKU	7KLB
Diffraction Source	MaNDi		Rigaku FR-E SuperBright	
Temperature (K)	296			
Space group	$P6_122$			
a, *b*, *c* (Å)	81.30, 81.30, 241.840	81.33, 81.33, 242.880	81.14, 81.14, 241.63	81.13, 81.13, 242.12
α, β, γ (°)	90, 90, 120			
Wavelengths (Å)	2-4		1.5418	
No. of images	8	8	180	280
Exposure time	48 h	48 h	60 s	60 s
No. of unique reflections	24556	21719	31815	25718
Total No. of reflections	196496	155817	248673	194364
Resolution range (Å)	14.64-2.20 (2.28-2.20)	14.65-2.30 (2.38-2.30)	50.00-2.02 (2.07-2.02)	50.00-2.16 (2.20-2.16)
Multiplicity	8.0 (6.1)	7.2 (5.7)	7.8 (3.5)	7.6 (4.1)
I/σ(I)	7.0 (3.40)	6.2 (3.3)	8.3 (2.0)	4.8 (2.0)
R_{merge}	0.284 (0.314)	0.277 (0.294)	-	-
R_{meas}	-	-	0.291 (0.610)	.459 (.683)
$CC_{1/2}$	0.935 (0.275)	0.943 (0.319)	0.950 (0.730)	0.804 (0.638)
R_{pim}	0.101 (0.129)	0.102 (0.124)	0.082 (0.320)	0.140 (0.326)
Data completeness (%)	98.83 (98.83)	98.94 (99.16)	100.0 (100.0)	97.5 (95.5)
Refinement Statistics				
R_{work}	0.2565	0.2493	0.2166	0.2075
R_{free}	0.2817	0.3021	0.2517	0.2544
No. of atoms	6606	6556	3291	3284
Protein including D	6228	6222	3162	3168
Solvent	376	332	127	98
Mn	2	2	2	2
R.m.s. deviations				
Bond lengths (Å)	0.093	0.092	0.002	0.006
Bond angles (°)	0.78	0.64	0.47	0.98
Average *B*-factor				
Protein	33.62	30.57	28.23	33.82
Water	30.51	29.19	30.64	31.91
Mn	25.3	23.53	17.00	24.30

Supplementary Methods

Data processing and refinement. X-ray refinement was performed by removing all non-protein entities in the starting model of 5VF9[1], simple molecular replacement through rigid-body refinement, and subsequent restrained-positional refinement. With *COOT*[2], the protein model was manually fit into $|F_o|$-$|F_c|$ peaks as needed and refined first. New solvent structure and Mn atoms were manually modeled into $|F_o|$-$|F_c|$ density. As D atoms were manually added during iterations of neutron refinement, the stereochemistry weight scale was manually adjusted due to the increased number of atoms at sensitive stereochemical positions. Loose Mn-coordination restraints were derived from the Mn^{2+}SOD X-ray structure and applied to the Mn^{2+}SOD neutron model, whereas the Mn^{3+}SOD neutron model used restraints derived from our own DFT calculations. In both cases, the R-free value was improved with the application of these restraints.

Computational Details. Computations from the NWChem 6.8 software package utilized an extra-fine integration grid quadrature known to provide high precision with restricted open-shell John-Sham (ROKS) treatment[3-5]. The geometry optimizations implemented the B3LYP exchange-correlation functional dispersion corrected according to Becke and Johnson damping (DFT-D3-BJ)[6,7]. Optimizations were first performed in the gas phase utilizing an energy convergence threshold of 1 E -6 atomic units. There was no notable difference when optimizations began directly in the solution phase other than longer computational times. The nearest three water molecules found in the neutron structure counterparts, representative of the ordered solvent found at the active site, were included in the QM models in addition to the Mn-ligated solvent

Solvation model. The COSMO solvation model treats solvent as an implicit dielectric continuum (i.e. many solvent molecules need not be explicitly modeled in the QM system). The charge distribution of the continuum is derived using a scaled-conductor boundary condition between the cavity surface and solvent[8]. The inclusion of several explicit solvent molecules in combination with an implicit solvent model to model explicitly known hydrogen bonds increases the accuracy of energy calculations[9]. In the case of the present QM system, the explicit water molecules utilized are representative of the ordered solvent found experimentally in the neutron structures and other published X-ray structures[1].

Supplementary References

1 Azadmanesh, J., Trickel, S. R. & Borgstahl, G. E. O. Substrate-analog binding and electrostatic surfaces of human manganese superoxide dismutase. *J. Struct. Biol.* **199**, 68-75, doi:10.1016/j.jsb.2017.04.011 (2017).

2 Emsley, P. & Cowtan, K. Coot: model-building tools for molecular graphics. *Acta Cryst. D* **60**, 2126-2132, doi:10.1107/S0907444904019158 (2004).

3 Papas, B. N. & Schaefer III, H. F. Concerning the precision of standard density functional programs: Gaussian, Molpro, NWChem, Q-Chem, and Gamess. *Comput. Theor. Chem.* **768**, 175-181 (2006).

4 Wheeler, S. E. & Houk, K. N. Integration Grid Errors for Meta-GGA-Predicted Reaction Energies: Origin of Grid Errors for the M06 Suite of Functionals. *J. Chem. Theory Comput.* **6**, 395-404, doi:10.1021/ct900639j (2010).

5 Valiev, M. *et al.* NWChem: A comprehensive and scalable open-source solution for large scale molecular simulation. *Comput. Phys. Commun.* **181**, 1477-1489 (2010).

6 Grimme, S., Ehrlich, S. & Goerigk, L. Effect of the damping function in dispersion corrected density functional theory. *J. Comput. Chem.* **32**, 1456-1465, doi:10.1002/jcc.21759 (2011).

7 Becke, A. D. Becke's three parameter hybrid method using the LYP correlation functional. *J. Chem. Phys.* **98**, 5648 (1993).

8 Klamt, A. Conductor-like Screening Model for Real Solvents: A New Approach to the Quantitative Calculation of Solvation Phenomena. *J. Phys. Chem.* **99**, 2224-2235 (1995).

9 Zhang, S., Baker, J. & Pulay, P. A reliable and efficient first principles-based method for predicting pK(a) values. 1. Methodology. *J. Phys. Chem. A* **114**, 425-431, doi:10.1021/jp9067069 (2010).
